# Neutralization of SARS-CoV-2 Variants by Serum from BNT162b2 Vaccine Recipients

**DOI:** 10.3390/v13102011

**Published:** 2021-10-06

**Authors:** Fabrizia Valleriani, Elisa Mancuso, Giacomo Vincifori, Liana Teodori, Lisa Di Marcantonio, Massimo Spedicato, Alessandra Leone, Giovanni Savini, Daniela Morelli, Barbara Bonfini, Alessio Lorusso

**Affiliations:** Istituto Zooprofilattico Sperimentale dell’Abruzzo e del Molise, 641000 Teramo, Italy; f.valleriani@izs.it (F.V.); e.mancuso@izs.it (E.M.); g.vincifori@izs.it (G.V.); l.teodori@izs.it (L.T.); l.dimarcantonio@izs.it (L.D.M.); m.spedicato@izs.it (M.S.); a.leone@izs.it (A.L.); g.savini@izs.it (G.S.); d.morelli@izs.it (D.M.); b.bonfini@izs.it (B.B.)

**Keywords:** SARS-CoV-2, neutralization, viruses, variants, cells, B.1.617.2 (Delta) variant

## Abstract

Severe acute respiratory syndrome coronavirus 2 (SARS-CoV-2) has evolved rapidly, leading to viral lineages characterized by multiple mutations in the spike protein, which could potentially confer to the virus the ability to avoid the vaccine-induced immune response, making the vaccines less effective or ineffective. Here, we initially evaluated the neutralization capabilities in vitro by serum neutralization (SN) of six serum samples collected from recipients of the BNT162b2 vaccine against 11 SARS-CoV-2 isolates belonging to the major SARS-CoV-2 lineages that had been circulating in Italy. Then, we considered 30 additional serum samples by SN assay against the dominant B.1.617.2 (Delta) variant. A B.1 lineage isolate was used as a reference. In the first analysis, significant differences when compared with the reference strain (*p* > 0.05) were not evidenced; instead, when the panel of 30 sera was tested against the B.1.617.2 (Delta) variant, a significant (*p* = 0.0015) 2.38-fold reduction in neutralizing titres compared with the reference after the first vaccine dose was demonstrated. After the second vaccine dose, the reduction was not significant (*p* = 0.1835). This study highlights that the BNT162b2 vaccine stimulates a humoral response able to neutralize all tested SARS-CoV-2 variants, thus suggesting a prominent role in mitigating the impact of the SARS-CoV-2 pandemic in real-world conditions. Long-term follow-up is currently ongoing.

## 1. Introduction

Severe acute respiratory syndrome coronavirus 2 (SARS-CoV-2), the zoonotic agent causative of the COVID-19 pandemic [1], has evolved rapidly, leading to several viral lineages that are nowadays circulating worldwide. Some of these lineages gained international concern for their potential to escape preventive and therapeutic countermeasures. These certainly include the recent Delta (PANGO lineage B.1.617.2) variant. This variant was first detected in India in December 2020 and was initially considered a variant of interest [2]. Given its rate of spread, it is considered a variant of concern (VOC) from May 2021. Currently, the B.1.617.2 (Delta) variant is by far the dominant SARS-CoV-2 variant in Italy (https://www.iss.it/documents/20126/0/Blettino+varianti+n.+9_17+settembre+2021.pdf/484b7aa2-2c0c-b109-4c31-087ed5c7b5af?t=1631890444760, last accessed 1 October 2021). The B.1.617.2 (Delta) variant is characterized by several spike protein mutations including L452R and P681R that could affect the immune responses [2], but current data on the COVID-19 vaccines efficacy against this variant are limited.

Before the emergence of the B.1.617.2 (Delta) variant, Italy faced several SARS-CoV-2 variants since the early spread of the pandemic virus [3], including two VOCs, Alpha and Gamma [4]. The Alpha variant (PANGO lineage B.1.1.7) was first detected in the United Kingdom in late September 2020 [2] and is characterized by 17 changes across the genome including the N501Y amino acid mutation and a two-amino acid deletion at positions 69 and 70 of the spike protein [5]. The Gamma variant (PANGO lineage P.1) was first identified in Brazil and shares two substitutions with the Beta (PANGO lineage B.1.351), E484K and N501Y, in the spike protein [2].

Several other less concerning variants were also detected by high-throughput sequencing by laboratories scattered all over the Italian territory [6]. These variants, all endowed with the spike D614G mutation, although less concerning as for their transmission and spread features, were poorly investigated upon the capabilities of serum samples of vaccinees to neutralize them.

Engineered vaccines are the most effective tools to mitigate the impact of the COVID-19 pandemic [7,8]. They act by stimulating the recipient immune system to produce specific antibodies that bind to the spike protein of SARS-CoV-2 and block the virus’ ability to infect the host cells. However, modifications in the protein sequence could potentially confer to the virus the ability to avoid the vaccine-induced immune response, making the vaccines less effective or ineffective against different SARS-CoV-2 variants. The impact of spike protein mutations of SARS-CoV-2 and their immunological role are still unclear. BNT162b2 (Comirnaty, Pfizer/BioNTech) vaccine was one of the first mRNA vaccines developed and the first to be listed by the WHO for the WHO Emergency Use Listing [9].

In this study, we evaluated the neutralization capabilities in vitro of serum samples collected from recipients of the BNT162b2 vaccine against isolates of the major SARS-CoV-2 variants circulating in Italy, including the last occurring B.1.617.2 (Delta) variant.

## 2. Materials and Methods

### 2.1. Ethical Approval

The human samples analyzed in this study were derived from the monitoring activities for SARS-CoV-2 antibodies performed by IZSAM on its employees. No ethical approval was specifically requested and written consent was obtained from each employee.

### 2.2. Sample Collection and Study Design

We tested a number of 36 sera that were available at the onset of the study and that had been collected from individuals, both males (*n* = 15) and females (*n* = 21), all health workers and first responders at IZSAM who had received two doses of the BNT162b2 mRNA vaccine (Comirnaty, Pfizer/BioNTech). The age of vaccinees ranged from 20–30 (N = 4), 31–40 (N = 9), 41–50 (N = 12), 51–60 (N = 9), and 61–70 (N = 2). These individuals did not report underlying health condition or risk factors. Serum samples had been collected 21 days after the first vaccine dose and 10 days after the second vaccine dose; they had been stored at −20 °C until testing. The set of sera was screened with two different ELISAs to detect antibodies against the spike (S) and nucleocapsid (N) proteins of SARS-CoV-2. All sera were evaluated in vitro for their neutralization capabilities against a reference isolate of the SARS-CoV-2 B.1 lineage, which is representative of the SARS-CoV-2 strains that had circulated in Italy during the first pandemic wave [3]. A subset of randomly selected sera from 6 individuals was tested by serum neutralization (SN) assay against isolates of different lineages of SARS-CoV-2, after the first and second vaccine dose. The remaining 30 sera were tested again by SN assay against the reference B.1 lineage and against the emerging B.1.617.2 (Delta) variant. One positive and one negative control serum were kindly provided by the Istituto Nazionale Malattie Infettive “Lazzaro Spallanzani” (INMI, Rome, Italy).

### 2.3. Cell Culture

The Grivet monkey (*Cercopithecus aethiops*) kidney epithelial cell line Vero E6 (C1008) was kindly provided by INMI. The cells were maintained in minimal essential medium (MEM, Sigma Aldrich, Merk Life Science S.r.l., Milan-Italy) supplemented with 10% fetal bovine serum (FBS, Sigma Aldrich, Merk Life Science S.r.l., Milan, Italy), 10^6^ IU/L penicillin, 10 g/L streptomycin, 5 × 10^6^ IU/L nystatin, and 125 mg/L gentamicin (IZSAM). The cell line was regularly checked for *Mycoplasma* contamination, and the absence was verified by PCR (Mycoplasma Detection Testing, Thermo Fisher, Waltham, MA, USA).

### 2.4. Virus Strains and Propagation

Twelve SARS-CoV-2 isolates at low cell passage were used in this study. A B.1 lineage isolate was used as the reference strain, while all other lineages were considered as new strains to be tested in SN. For greater clarity, all strains are identified by the virus name and GISAID accession number (Table 1). SARS-CoV-2 strains had been identified first by qRT-PCR on human nasopharyngeal swabs, as previously described [9], and then by high-throughput sequencing to obtain the whole genome sequence, as already reported by our group [6]. Swabs were selected for viral isolation based on sequence analysis, and lineages of interest had been isolated on Vero E6 cells under biosafety level 3 (BSL-3) conditions in the biocontainment laboratory at the IZSAM.

All 12 isolates were propagated in Vero E6 cells using MEM supplemented with 10% FBS. Cells were seeded in 175 cm^2^ flasks at 10^6^ cells/mL and after 24 h were infected with 5 mL of a viral suspension at 0.01 multiplicity of infection. The flasks were incubated at 37 °C in a humidified atmosphere of 5% CO_2_ and observed daily under an inverted optical microscope. When cytopathic effect (CPE) affected 80–90% of the cell monolayer, the supernatant was collected and centrifuged at 4 °C 2000 rpm for 10 min to remove the cellular pellet. Then, the supernatant was aliquoted and stored at −80 °C. Before use, the virus was titrated in serial 1 log dilutions (from 1 log to 8 log) in 96-well culture plates of Vero E6 cells to determine the 50% tissue culture infective dose (TCID_50_). Plates were incubated at 37 °C and checked every day to identify CPE using an inverted optical microscope. The endpoint titres were calculated according to the Reed and Muench method based on 10 replicates for titration [10].

### 2.5. Serum Neutralization Assay

We used the SN assay as previously described [11] to determine the neutralization activity of vaccine-induced antibodies. Before testing, serum samples were inactivated by heating at 56 °C for 30 min. Two-fold serial dilutions (from 1:10 to 1:1280) of the tested sera and the positive and negative control sera were prepared in 96-well plates using MEM supplemented with 2% FBS. Subsequently, an equal volume of 100 TCID_50_/mL of B.1 isolate was added to the diluted serum samples, and plates were incubated for 30 min at 37 °C in 5% CO_2_. After incubation, the serum–virus solutions were transferred to 96-well plates containing confluent Vero E6 cells seeded the day before. These plates were incubated for 72 h at 37 °C in 5% CO_2_ and were observed using an inverted microscope for a virus-specific CPE. The neutralization titre was defined as the reciprocal of the highest dilution without any CPE in the wells.

### 2.6. Enzyme-Linked Immunosorbent Assays

Two different enzyme-linked immunosorbent assay (ELISA) kits were used to detect specific antibodies against the SARS-CoV-2 S and N proteins in human serum samples. IgG antibodies against the spike protein S1 receptor-binding domain (S1 RBD) were detected with the SARS-CoV-2 S1 RBD IgG ELISA kit (cat: MBS398005; MyBiosource, San Diego, CA, USA). Test results for each sample were calculated as the ratio between the optical density (OD) of the serum sample and OD of the positive control and were expressed as a percentage. Values ≥ 45% were considered positive. Instead, specific IgG antibodies binding to SARS-CoV-2 N protein were determined using the ERADIKIT COVID19-IgG (cat: 26843-05; In3diagnostic, Turin, Italy). The results were defined based on the calculated ratio described in the following formula and expressed as percentage:

PR (%) = (OD test sample − OD negative control)/(OD positive control − OD negative control).

Values ≥ 40% were considered positive for the presence of antibodies against SARS-CoV-2.

### 2.7. Statistical Analysis

Comparison of neutralizing antibody titres by SN in the selected 6 serum samples against SARS-CoV-2 variants was performed by comparing the mean of each group with the mean of the control group (B.1) by one-way analysis of variance (ANOVA) followed Holm–Šídák’s multiple comparisons test (between groups). Comparison of neutralizing antibody titres by SN in 30 serum samples against B.1 and B.1.617.2 (Delta) was performed with the non-parametric Mann–Whitney test. *p* values < 0.05 were considered statistically significant. Analyses and graphs were obtained with GraphPad Prism 9 software (v9.0, La Jolla, CA, USA). Mean titres are expressed throughout the manuscript as the reciprocal of the highest serum dilution able to inhibit the virus cytopathic effect ± standard error (SEM).

## 3. Results

Serum samples from 36 BNT162b2-vaccinees were screened by ELISA and by SN, against a reference SARS-CoV-2 isolate, to evaluate the serological response after the two vaccine doses (Table 2). After the first dose, 35 of the 36 recipients showed neutralizing antibodies to the B.1 variant isolate that were detectable with the SN assay, while antibodies against the S protein by ELISA were detected in only 27 of the 35 SN-positive sera. After the second vaccine dose, all samples were positive on both the SN assay and S protein ELISA. Instead, antibodies against the N protein were not detected either after the first or second vaccine dose, suggesting that all 36 individuals had not previously been infected by wild-type SARS-CoV-2.

**Figure 1:**
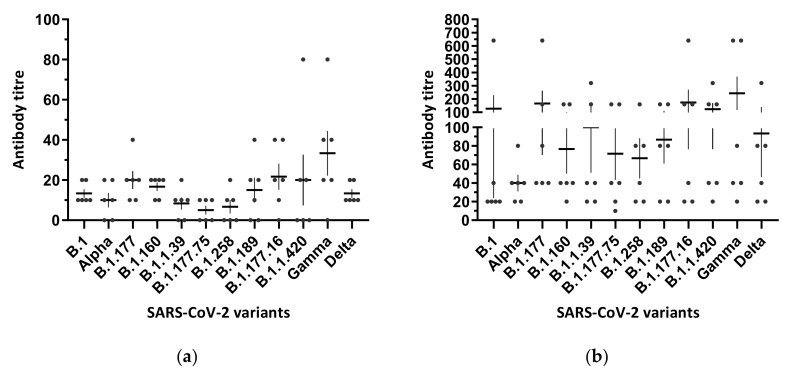
Antibody titres measured by serum neutralization assay against 11 SARS-CoV-2 isolates representative of the major lineages identified in Italy. (**a**) Serum samples of six vaccinees collected 21 days after the first vaccine dose. (**b**) Serum samples of the same six vaccinees collected 10 days after the second vaccine dose. Each dot represents one tested serum sample. Antibody titre is plotted as the reciprocal of the highest serum dilution able to inhibit the virus’ cytopathic effects on Vero E6 cells. Serum donors had been vaccinated with BNT162b2 (Comirnaty, Pfizer/BioNTech). One-way analysis of variance (ANOVA) followed Holm–Šídák’s multiple comparisons test was made to compare the mean of each group with the mean of the control group (B.1). All comparisons vs. the reference control group gave *p* > 0.05. Figures were obtained by GraphPad Prism 9 software (La Jolla, CA 92037, USA).

Thus, we focused on SARS-CoV-2 lineages that have been circulating in Italy since the beginning of the pandemic. In order to do so, the neutralization capabilities of sera from six vaccinees after the first and second BNT162b2 vaccine doses were tested against the B.1 lineage of SARS-CoV-2, which was used as a reference, and another 11 SARS-CoV-2 lineages, by SN assay. After the first BNT162b2 dose, vaccine-induced antibodies against the reference strain were detected in all sera (mean 13.33 + 2.108; Figure 1a). The titres ranged between 0 and 80 when the sera were tested against the other viral lineages, with mean values between five and 33.3 across all lineages. As for the major VOCs, the B.1.17 (Alpha) variant isolate showed a mean 1.30-fold reduction (mean 10 ± 3.65) with respect to the B.1 reference isolate when tested against the six serum samples collected after the first vaccine dose, whereas fold reduction was not evidenced against the B.1.617.2 (Delta) variant isolate (mean 13.33 ± 2.10). As opposite, the reference B.1 isolate showed a mean 2.5-fold reduction with respect to the mean (33.33 ± 11.16) titre of the P.1 (Gamma) variant isolate. Nevertheless, in all cases, differences were not statistically significant.

After the second vaccine dose, all six sera tested against the reference lineage isolate showed neutralizing vaccine-induced antibodies (Figure 1b). The neutralization titres were higher than those observed in all six vaccinees after the first dose and ranged between 20 and 640 (mean 126.7 ± 102.7). As for the major VOCs, a 3.16-fold and a 1.35-fold reduction were evidenced against the B.1.17 (Alpha) (mean 40 ± 8.94) and B.1.617.2 (Delta) variant (mean 93.33 ± 46.67) isolates, respectively. The reference B.1 isolate showed reductions, 1.9-fold, 1.31-fold, and 1.3-fold, with respect to the mean titre of the P.1 (Gamma) variant isolate (mean 243.3 ± 125.7), the B.1.177 variant isolate (mean 166.7 ± 96.56), and the B.1.177.16 variant isolate (mean 173.3 ± 97.11), respectively. As for the remaining variant isolates, a reduction in neutralizing titres compared with the B.1 virus in vaccinated individuals was evidenced for variant B.1.160 (1.65-fold, mean 76.67 ± 26.54), B.1.1.39 (1.26-fold, mean 100 ± 48.99), B.1.177.75 (1.76 fold, mean 71.67 ± 28.33), B.1.258 (1.9-fold, mean 66.67 ± 21.71), B.1.189 (1.46-fold, mean 86.67 ± 25.65), and B.1.1.420 (1.02-fold, mean 123.3 ± 46.88). In all cases, neutralization titres obtained using the above-mentioned 11 lineages did not show any significant differences when compared pairwise with the reference strain (*p* > 0.05).

**Figure 2:**
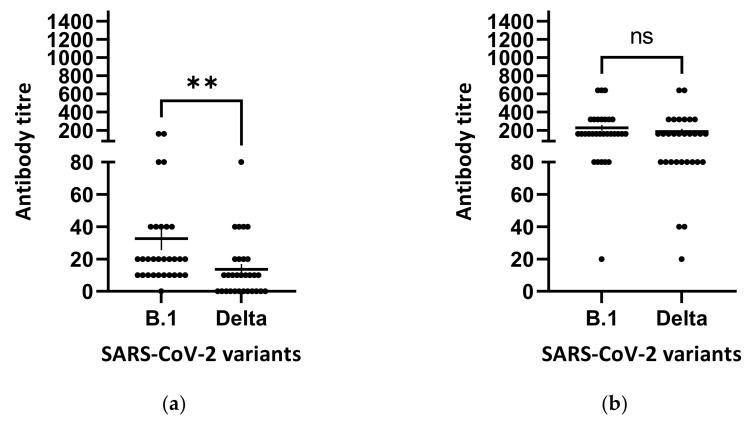
SARS-CoV-2 neutralizing antibody titres against SARS-CoV-2 B.1. and B.1.617.2 isolates measured by serum-neutralization. The mean values and standard error bars are presented. Serum samples of vaccinees collected 21 days after the first vaccine dose (**a**). Serum samples of the same vaccinees collected 10 days after the second vaccine dose (**b**). Antibody titre is plotted as the reciprocal of the highest serum dilution able to inhibit the virus cytopathic effect. Each dot represents one tested serum sample. Serum donors were vaccinated with BNT162b2 (Comirnaty, Pfizer/BioNTech mRNA vaccine). Comparison of neutralizing antibody titres by SN in 30 serum samples against B.1 and B.1.617.2 (Delta) was performed with the non-parametric Mann–Whitney test; ** *p* = 0.0015. ns, non-significant. Figures were obtained by GraphPad Prism 9 software (La Jolla, CA 92037, USA).

Thus, although the mean of neutralization titres against the B.1.617.2 (Delta) after the second vaccine dose was not statistically different from that obtained with the reference isolate, we decided to test by SN all batches of serum samples (N = 30) against this emerging and dominant variant in Italy (https://www.iss.it/documents/20126/0/Bollettino+varianti+n.+9_17+settembre+2021.pdf/484b7aa2-2c0c-b109-4c31-087ed5c7b5af?t=1631890444760, last accessed 1 October 2021) as in other parts of the world. After the first vaccine dose (Figure 2a), the neutralization titres had a mean of 13.67 ± 3.33 when sera were tested against the B.1.617.2 (Delta) lineage isolate. Indeed, differences with the control group (B.1 lineage isolate, mean 32.67 ± 7.19) were statistically significant (2 tailed *p* = 0.0015) with a 2.38-fold reduction. After the second vaccine dose (Figure 2b), serum samples against the B.1.617.2 (Delta) lineage showed a mean titre of 187.3 ± 28.32. In pairwise comparison with the control group (mean 227.3 ± 30.06), no difference, with a 1.21-fold reduction, was evidenced (two-tailed *p* = 0.1835).

## 4. Discussion

Recent data suggest that vaccination with BNT162b2 is effective against the B.1.1.7 (Alpha), B.1.351 (Beta), P.1 (Gamma), and B.1.617.2 (Delta) VOCs, albeit at different degrees [12,13,14,15,16]. We here demonstrate that neutralizing levels against both B.1.617.2 (Delta) isolates were significantly reduced only after the first dose of BNT162b2 vaccine with respect to the reference B.1 lineage isolate. Indeed, after the second dose of vaccine, the mean titre against the B.1.617.2 (Delta) variant isolate was only slightly reduced with respect to the reference strain but without any statistical difference. This would suggest that BNT162b2 vaccination is also probably protective in vivo against the B.1.617.2 (Delta) VOC, at least 10 days after the second vaccine dose. We also studied the neutralization capabilities of serum samples collected from recipients of the BNT162b2 vaccine against isolates of the major SARS-CoV-2 lineages circulating in Italy, including the B.1.1.7 (Alpha) and P.1 (Gamma) variants but also against less concerning (and less investigated) variants such as B.1.160, B.1.1.39, B.1.177.75, B.1.258, B.1.189, B.1.177.16, and B.1.1.420. Overall, following the second vaccine dose, serum samples of vaccinees were able to neutralize efficiently all SARS-CoV-2 variant isolates used in this study. In a few occasions (B.1.177 and P.1-Gamma variants), the mean of antibody titres was even higher than that of the reference B.1 lineage strain. As for the gamma variant, our results are in stark contrast with previous studies [17] that found a significant loss of neutralizing activity against P.1 (Gamma) lineage in sera from BNT162b2 vaccinees. Definitely, in this perspective, additional experiments are warranted.

We used a B.1 isolate [18] as a reference, as it was the only isolate which showed only one unique missense mutation (Spike D614G) all over the viral genome and therefore considered the most related virus to the prototype WIV04 SARS-CoV-2 strain with hCoV-19/Wuhan/WIV04/2019 as the official reference sequence employed by GISAID (EPI_ISL_402124). 

The data presented here contribute to the growing evidence of effectiveness of the mRNA-based BNT162b2 vaccine against known VOCs and highlight the importance of vaccination specifically in areas with high proportion of VOCs circulation. The strengths of this study are the use of wild-type low-passaged isolated viruses and not mutated pseudo-viruses to evaluate the full neutralizing response and the study of neutralization of less investigated SARS-CoV-2 variants. The limitations of the study include the small number of sera analyzed, the lack of T-cell response evaluation, the lack of other relevant SARS-CoV-2 variants such as the beta and the theta variants, and the lack of multiple isolates for each variant. Importantly, we only measured neutralizing antibodies at the peak after the vaccination schedule. Accordingly, the measurement of antibodies from serum samples of the same vaccinees collected after months is currently ongoing. This would certainly help to evaluate the duration of humoral response induced by the BNT162b2 vaccine over time. We also acknowledge that a larger number of serum samples was tested only against the reference isolate and the B.1.617.2 (Delta) variant, as this latter is the most worrisome, emerging, and by far dominant variant threating the public health systems of several countries and of major concern for the massive vaccination campaign.

Overall, our results suggest that despite somewhat reduced neutralization capacity after the first vaccine dose, BNT162b2 vaccination induces a substantial antibody response also for the B.1.617.2 (Delta) VOC. Further studies are necessary to confirm the vaccine effectiveness in broader population groups and the duration of humoral immunity over time.

## Figures and Tables

**Table 1:** Viral isolates used in the study.

PANGO Lineage ^1^	Virus Name	GISAID Acc. no.	Spike Mutations
B.1	hCoV-19/Italy/ABR-IZSGC-TE46419/2020	EPI_ISL_529023	D614G
B.1.177.83	hCoV-19/Italy/ABR-IZSGC-351570/2020	EPI_ISL_806743	A222V, A262S, D614G, L452R, P272L
B.1.1.7 (Alpha)	hCoV-19/Italy/ABR-TE353967/2020	EPI_ISL_738046	A570D, D614G, D1118H, H69del, N501Y, P681H, S982A, T716I, V70del, Y144del
B.1.160	hCoV-19/Italy/ABR-IZSGC-91401/2021	EPI_ISL_1117522	D614G, S477N
B.1.1.39	hCoV-19/Italy/ABR-IZSGC-353326/2020	EPI_ISL_806746	D614G
B.1.177.75	hCoV-19/Italy/ABR-IZSGC-TE362018/2020	EPI_ISL_833085	A222V, D614G,
B.1.258	hCoV-19/Italy/ABR-IZSGC-101439/2021	EPI_ISL_1250984	A688V, D614G, N439K
B.1.177	hCoV-19/Italy/ABR-IZSGC-100865/2021	EPI_ISL_1117525	D614G, N501Y
B.1.177.81	hCoV-19/Italy/ABR-IZSGC-91677/2021	EPI_ISL_1117524	A222V, D614G, S71F
B.1.1.420	hCoV-19/Italy/ABR-IZSGC-98432/2021	EPI_ISL_1117698	D614G, L18F, N440K, P26S, Q677H
P.1 (Gamma)	hCoV-19/Italy/ABR-IZSGC-106022/2021	EPI_ISL_1251006	D138Y, D614G, E484K, H655Y, K417T, L18F, N501Y, P26S, P1112Q, R190S, T20N, T1027I, V1176F
B.1.617.2 (Delta)	hCoV-19/Italy/ABR-IZSGC-288699/2021	EPI_ISL_2673654	D614G, D950N, E156G, F157del, L452R, P681R, R158del, T19R, T274I, T478K

^1^ Lineage version 28 July 2021. B.1 lineage used as reference strain. Acc. no.: Accession number. GISAID: Global Initiative on Sharing Avian Influenza Data. PANGO: Phylogenetic Assignment of Named Global Outbreak.

**Table 2:** ELISA for antibodies against the spike and nucleocapsid proteins, after the first and second vaccine doses, of the 36 serum samples tested by SN.

Test Result	First Dose			Second Dose		
	SN	Spike ELISA	Nucleocapsid ELISA	SN	Spike ELISA	Nucleocapsid ELISA
**Positive**	35	27	0	36	36	0
**Negative**	1	9	36	0	0	36

SN, serum neutralization assay with a B.1 isolate.

## Data Availability

Sequence data are available via GISAID. The accession numbers for the sequences used can be found in Table 1.
